# Comparative genomics of the coconut crab and other decapod crustaceans: exploring the molecular basis of terrestrial adaptation

**DOI:** 10.1186/s12864-021-07636-9

**Published:** 2021-04-30

**Authors:** Werner Pieter Veldsman, Ka Yan Ma, Jerome Ho Lam Hui, Ting Fung Chan, J. Antonio Baeza, Jing Qin, Ka Hou Chu

**Affiliations:** 1 School of Life Sciences, The Chinese University of Hong Kong, Shatin, Hong Kong SAR China; 2 Department of Biological Sciences, Clemson University, 132 Long Hall, Clemson, SC 29634 USA; 3 Smithsonian Marine Station at Fort Pierce, 701 Seaway Drive, Fort Pierce, Florida 34949 USA; 4 Departamento de Biología Marina, Facultad de Ciencias del Mar, Universidad Católica del Norte, Larrondo, 1281 Coquimbo, Chile; 5 School of Pharmaceutical Sciences (Shenzhen), Sun Yat-sen University, Shenzhen, China

**Keywords:** *Birgus latro*, Nuclear genome, *Panulirus ornatus*, *Paralithodes camtschaticus*

## Abstract

**Background:**

The complex life cycle of the coconut crab, *Birgus latro*, begins when an obligate terrestrial adult female visits the intertidal to hatch zoea larvae into the surf. After drifting for several weeks in the ocean, the post-larval glaucothoes settle in the shallow subtidal zone, undergo metamorphosis, and the early juveniles then subsequently make their way to land where they undergo further physiological changes that prevent them from ever entering the sea again. Here, we sequenced, assembled and analyzed the coconut crab genome to shed light on its adaptation to terrestrial life. For comparison, we also assembled the genomes of the long-tailed marine-living ornate spiny lobster, *Panulirus ornatus*, and the short-tailed marine-living red king crab, *Paralithodes camtschaticus*. Our selection of the latter two organisms furthermore allowed us to explore parallel evolution of the crab-like form in anomurans.

**Results:**

All three assembled genomes are large, repeat-rich and AT-rich. Functional analysis reveals that the coconut crab has undergone proliferation of genes involved in the visual, respiratory, olfactory and cytoskeletal systems. Given that the coconut crab has atypical mitochondrial DNA compared to other anomurans, we argue that an abundance of *kif22* and other significantly proliferated genes annotated with mitochondrial and microtubule functions, point to unique mechanisms involved in providing cellular energy via nuclear protein-coding genes supplementing mitochondrial and microtubule function. We furthermore detected in the coconut crab a significantly proliferated HOX gene, *caudal*, that has been associated with posterior development in *Drosophila*, but we could not definitively associate this gene with carcinization in the Anomura since it is also significantly proliferated in the ornate spiny lobster. However, a cuticle-associated coatomer gene, *gammacop*, that is significantly proliferated in the coconut crab, may play a role in hardening of the adult coconut crab abdomen in order to mitigate desiccation in terrestrial environments.

**Conclusion:**

The abundance of genomic features in the three assembled genomes serve as a source of hypotheses for future studies of anomuran environmental adaptations such as shell-utilization, perception of visual and olfactory cues in terrestrial environments, and cuticle sclerotization. We hypothesize that the coconut crab exhibits gene proliferation in lieu of alternative splicing as a terrestrial adaptation mechanism and propose life-stage transcriptomic assays to test this hypothesis.

**Supplementary Information:**

The online version contains supplementary material available at 10.1186/s12864-021-07636-9.

## Background

All terrestrial plants and animals evolved directly or indirectly from life in the ocean. Land plants, that arose from an ancestral terrestrialization event within charophytic algae [1], colonized terrestrial environments earlier than animals. In the case of vertebrates, evidence points to a single land colonization event (with some subsequent reversions to the aquatic environment) [2], while in the invertebrates, there were multiple crossings of the water-land barrier within distantly related clades including the Mollusca [3] and Arthropoda [2]. Ancient terrestrialization events within the Arthropoda are known to have occurred in the Hexapoda, Myriapoda and Arachnida. Further terrestrialization events within the malacostracan crustaceans are considered to be some of the most recent evolutionary crossings of the water-land barrier [2]. The coconut crab, *Birgus latro*, is an example of such a recently terrestrialized member of the Malacostraca. The complex life cycle of a coconut crab begins with a newly hatched larva being cast into the ocean at high tide by its maternal parent. If it survives the zoeal stage adrift in the ocean, it settles to the bottom in the shallow subtidal zone. The newly metamorphosed post-larval glaucothoe then utilizes an empty gastropod shell for protection and migrates to the coastline with the shell on its back [4], never to return to the sea again other than for spawning in the case of females. Aquatic-to-terrestrial migratory arthropods such as the coconut crab have to be able to adapt to life in both water and on land. It furthermore follows that the coconut crab’s genomic, physiological, and morphological characteristics must be different from both fully aquatic decapods such as the closely related *Paralithodes* species and fully terrestrial malacostracans such as some members of the Isopoda and Amphipoda. We predict then that a life cycle that involves both aquatic and terrestrial life stages would require the coconut crab to undergo a change in its genomic product complement as it crosses the boundary between sea and land. Based on the notion that biochemical energy conservation is a trait under universal selection (as discussed in [5]), it can be inferred that any advantages that an organism’s genomic constitution confers upon it specifically to cope with an aquatic environment, would become redundant and therefore an energy burden once the organism transits to land. The coconut crab would accordingly be in need of genomic flexibility brought about by a dynamic process that shifts the equilibrium of its genomic products from an aquatic to terrestrial optimized complement for the purpose of energy conservation once the coconut crab permanently leaves the aquatic environment for the terrestrial environment.

To investigate the phenotype of compulsory terrestrialism in the coconut crab, we have assembled and annotated the genomes of two anomurans: the coconut crab (*B. latro*) and the marine-living red king crab (*Paralithodes camtschaticus*). Moreover, to provide context to study the crab-like morphotype in the Anomura, we have assembled and annotated the genome of the long-tailed marine-living ornate spiny lobster, *Panulirus ornatus*. The assembly of these three genomes will greatly contribute to comparative genomics research by providing a plethora of molecular markers for use in functional and comparative genomic studies that may, for example, answer questions related to shell-utilization, perception of visual and olfactory cues, and cuticle sclerotization in the Anomura. Our results in specific show that compared to eight other malacostracans, the coconut crab has undergone proliferation in several genes associated with the visual, respiratory, olfactory and cytoskeletal systems. The adult coconut crab also has muted alternative splicing compared to three obligate aquatic decapods. In a previous study, we reported that the coconut crab has mutated mitochondrial tDNAs compared to other anomurans [6] and we now observe mitochondrial targeting signals within genes annotated with mitochondrial and microtubule function, most notably, in a massively proliferated kinesin. We therefore propose a testable hypothesis postulating that lowered alternative splicing coupled with proliferated genes that are annotated with functions that overlap with those of tissues where lowered alternative splicing is observed, confer upon the coconut crab the ability to adapt to its changing environment. In conclusion we recommend the design of transcriptomic assays that include both temporal and spatial aspects to test this hypothesis. Our expectation is that such a study would reveal whether the coconut crab displays higher alternative splicing during its early life in an aquatic environment.

## Results

### The newly assembled genomes are large, AT-rich and repetitive

The estimated genome sizes of the coconut crab (6.22 Gbp) and red king crab (7.29 Gbp) are each about twice the size of the spiny lobster genome (3.23 Gbp, Table 1). Although BUSCO analysis resulted in detection of about 90% (complete and fragmented) signature arthropod homologs in each of the assemblies, the assembly sizes for each of the three organisms are about half of the estimated genome sizes. The source of this discrepancy is not clear but could possibly be the result of genomic ambiguity introduced by repetitive elements. The low contig N50 values of between 5 kbp and 6 kbp were only marginally improved upon by gap-filling the assemblies using Illumina paired-end short-reads. The spiny lobster scaffold N50 has the best post gap-filling improvement of 8.1 kbp. The *Panulirus ornatus* assembly contains 403,948 scaffolds, the *B. latro* assembly 767,271 scaffolds and the *Paralithodes camtschaticus* assembly 859,965 scaffolds. These scaffold numbers are inversely associated with the amount of linked-read data that were generated for the three species (two lanes of linked-read data for the red king crab, three for the coconut crab, and eight for the spiny lobster). The inverse relationship suggests that more contiguous genomes might be generated by additional linked-read sequencing and that long read sequencing [7, 8] may be a prudent choice. All three genomes are highly repetitive with classified interspersed repeats taking up 14.13% of the *Panulirus ornatus* genome, 23.81% of the *B. latro* genome, and 26.65% of the *Paralithodes camtschaticus* genome (Table 2).

**Table 1:** Summary statistics on genome assembly, genome completeness and AT-content

Organism	Estimated genome size (Gbp)	Assembly size (Gbp)	Contig N50 (bp)	Scaffold N50 (bp)	Scaffolds larger than 100 Kbp	Fragmented signature homologs (%)	Complete signature homologs (%)	AT-content of called bases (%)
*Birgus latro*	6.22	2.96	5342	6350	1054	23.2	63.5	57.56
*Panulirus ornatus*	3.23	1.93	5451	8144	1787	15.5	77.6	57.36
*Paralithodes camtschaticus*	7.29	3.81	5815	7037	637	29.5	57.6	58.77

**Table 2:** Percentage repetitive elements in the assembled genomes

Repeat type	*Birgus latro*	*Paralithodes camtschaticus*	*Panulirus ornatus*
DNA elements	4.36	3.70	1.04
LINEs	15.27	15.34	12.12
Low complexity	0.70	0.29	0.19
LTR elements	3.97	6.81	0.84
Satellites	0.06	0.03	0.03
Simple repeats	4.96	2.68	2.73
SINEs	0.21	0.80	0.13
Small RNAs	0.02	0.09	0.02
Unclassified	29.80	38.56	24.42
	59.35	68.30	41.52

Long interspersed nuclear elements (LINEs) are the most numerous of the interspersed elements in all three assembled genomes with short interspersed nuclear elements (SINEs) being most numerous in *Paralithodes camtschaticus*. The number of long terminal repeats (LTRs) are notably different in all three species. The genomes furthermore reflect a bias toward AT-content with the percentage AT-content of called bases being remarkably similar within the narrow range of 57.36 to 58.77%.

Ab initio gene prediction resulted in the detection of 23,818 complete coding sequences in *B. latro*, 28,597 in *Paralithodes camtschaticus*, and 99,127 in *Panulirus ornatus*. The value of using RNA-seq data during structural annotation is emphasized in Table S1 (Additional file 1), which shows that RNA-seq assisted annotation (with Augustus UTR training) greatly promotes the discovery of contained and overlapping coding genes in all three species. Predicted non-coding transfer RNA (tRNA) genes are most numerous for *Panulirus ornatus*, followed by *Paralithodes camtschaticus* and then *B. latro* (Table S2, Additional file 2). The glycine carrying tRNA with anticodon *gcc* is a notable exception where *B. latro* has a substantially larger number of copies than its counterparts.

### Comparative genomics and phylogenetic congruence with current systematic status

Clustering of all Eggnog predicted homologs into their best fitting taxa results in 12 taxonomic groupings across the nine malacostracan species under comparison (Table S3, Additional file 3). As expected, orthologs mostly clustered under Arthropoda, followed by the Metazoa and Eukaryota. Classification under bacteria is both consistent and low in number across the compared species, which indicates that bacterial contamination is at acceptable levels for all assemblies. The two king crab assemblies have a nearly identical number of orthologs clustered under Arthropoda despite the *Paralithodes camtschaticus* assembly being two orders of magnitude more fragmented than the *Paralithodes platypus* assembly. The latter genome, however, has three times as many orthologs clustered under the more generic metazoan taxa. Functional annotation of orthologous groups predicted from RNA-seq based annotation reveals that *Paralithodes camtschaticus* has the highest number of orthologs in most functional categories, particularly in carbohydrate/nucleotide metabolism and transport as well as in the central dogma categories of replication, transcription and translation (Table S4, Additional file 4). The coconut crab shows gene proliferation in the cytoskeletal related category, while in the spiny lobster, coenzyme metabolism is the only category with higher proliferation than in the other two species.

**Fig. 1:**
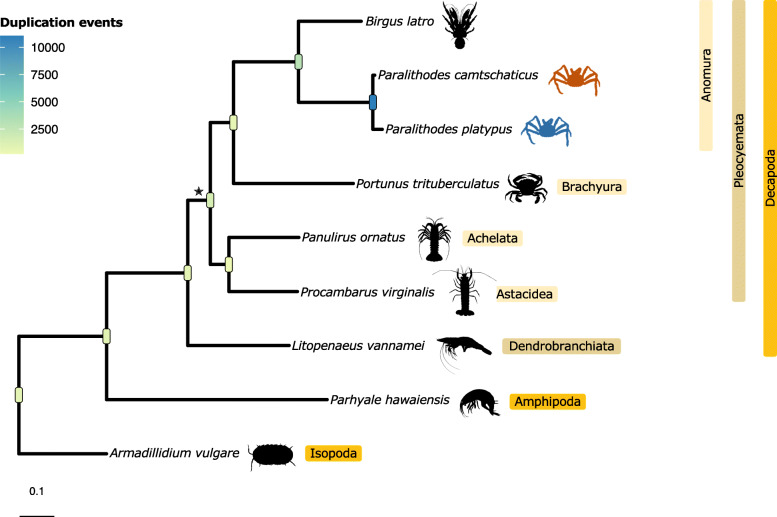
Phylogeny of the compared species. Interleave nodes on the tree are color coded with observed duplication events. All branches have 100% bootstrap support (separately determined with a maximum likelihood approach using 40 single copy orthologs) unless otherwise indicated with a star. This figure was drawn with ggtree version 2.2.3 [9] and Microsoft PowerPoint

**Fig. 2:**
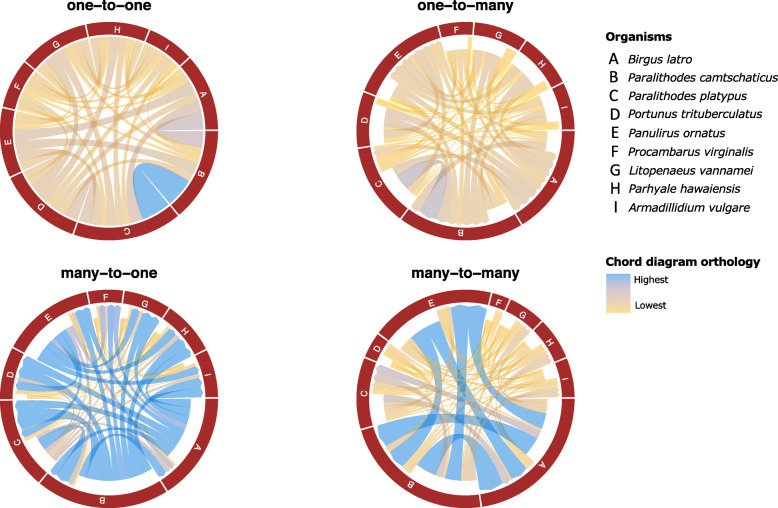
Orthological relationships between the compared genomes. Shared orthologs are placed into four cardinal groups. **a** one-to-one orthology **b** one-to-many orthology **c** many-to-one orthology, and **d** many-to-many orthology. This figure was drawn with Circlize version 0.4.10 [10] and Microsoft PowerPoint

Phylogenetic analysis using 40 single copy orthologs (Table S5, Additional file 5) that were detected by Orthofinder results in a well-supported phylogenic tree with relationships consistent with the current systematic status of the nine species (Fig. 1). Plotting the cardinality of orthologous relationships detected by Orthofinder shows, as expected, that the two king crabs have the highest number of one-to-one orthologs (Fig. 2). This latter result is in line with the similarity in arthropodan orthology between the two species, and reciprocally validates the completeness (not the contiguity) of these two genomes that were assembled by different research teams. One-to-many cardinality reveals highest orthology from single orthologs in *Paralithodes platypus* with multiple orthologs in *Paralithodes camtschaticus*. Many-to-one and one-to-many cardinality shows the highest number of directional orthology for the three anomurans under study and *Panulirus ornatus*, which follows the general increase in the number of gene duplications observed in phylogenetic divergence towards the Lithodidae.

### Mitochondrial targeting motifs

**Fig. 3:**
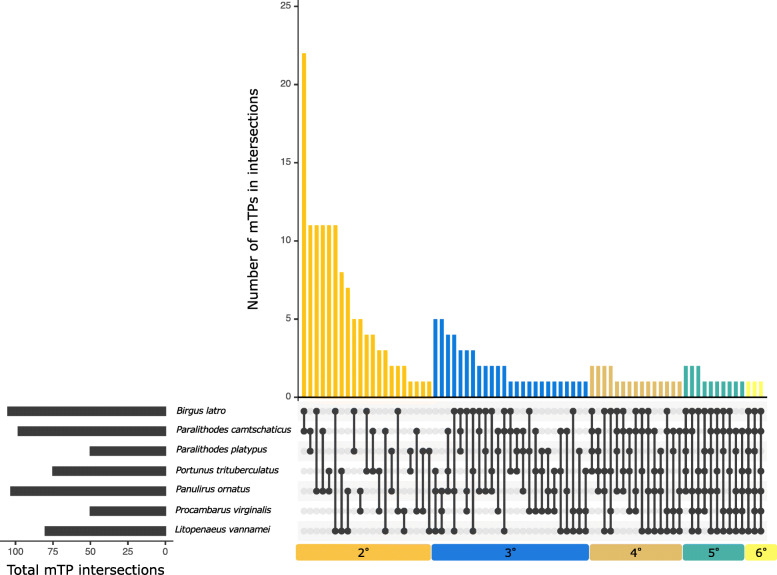
Nuclear expressed mitochondrial-targeting protein (mTP) interaction. Genes that contain mTP signals are shown in this interaction plot, which is similar in concept to a Venn-diagram. Degrees refer to the number of sets that have a given number of features in common. It is worth noting that the mTP-signal containing gene with the highest number of copies, *kif22*, is most proliferated in *B. latro*. This figure was drawn with UpSetR version 1.4.0 [11] and Microsoft PowerPoint

Scanning nuclear protein-coding genes for mitochondrial targeting signals across the seven decapods under study resulted in the most proteins with mitochondrial signals (mTPs) being found in *Paralithodes camtschaticus* (Fig. 3). Interestingly, *Paralithodes platypus* has less mTPs than *Portunus trituberculatus* and *Litopenaeus vannamei*, suggesting that the high number of mTPs of *Paralithodes camtschaticus* are isomorphs revealed by RNA-seq assisted annotation. Despite *Paralithodes camtschaticus* having the most unique mTPs, the mTP gene with the highest number of copies is the proliferated *kif22* gene in *B. latro*.

### Alternative splicing and gene proliferation

**Fig. 4:**
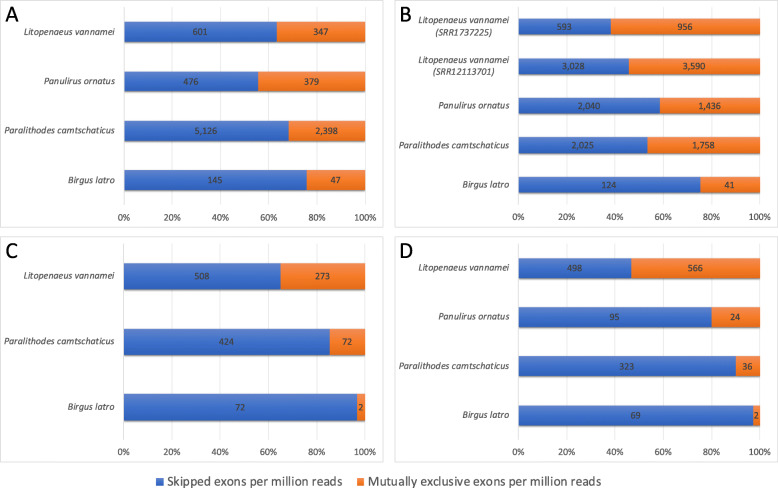
Proportional representation of alternative splicing profiles. The ratio of skipped exons to mutually exclusive exons are represented as percentage contribution with respect to their combined occurrence. Values within the bars indicate the number of alternatively spliced constructs. Each assayed tissue type is represented by an individual plot: **a** eyestalk, **b** gill, **c** hepatopancreas and **d** muscle. Identifiers starting with SRR are Sequence Read Archive (SRA) identifiers. This graph was drawn with Microsoft Excel

**Fig. 5:**
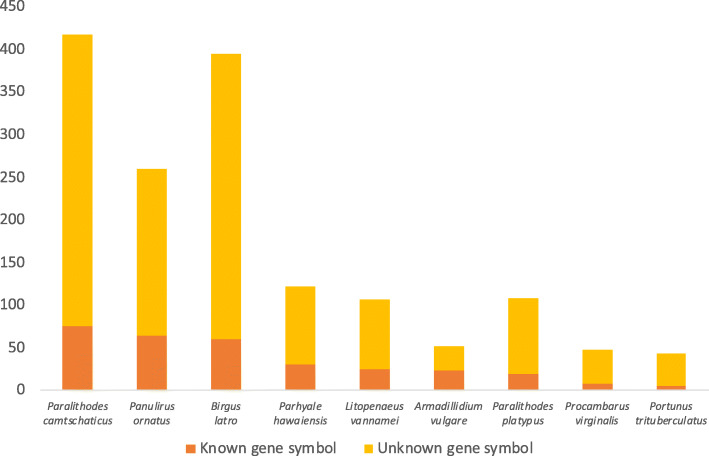
Comparison of coding sequences containing more than 25% SR-content. The genomes of the three species assembled in this study have the highest overall proportion of coding sequences with more than 25% serine/arginine (SR) content, while the two anomurans have a disproportionate number of high SR-content coding sequences without annotated gene symbols. This graph was drawn with Microsoft Excel

All three species under study (as well as the Pacific white shrimp that was included for comparison) have genes under alternative splicing in all assayed tissues (Fig. 4). Only these four decapod species were compared because transcriptomic data for *Paralithodes platypus*, *Portunus trituberculatus*, and *Procambarus virginalis* were either not available or did not cover all four tissue types of interest. Stringent filtering of the Outrigger output to retain only predicted splice junctions that have at least 10 forward and 10 reverse reads mapped to a given junction, and constructs that have a percent spliced in (PSI) value exceeding 0.05, reveals that *B. latro* exhibits lower absolute and reads-per-million adjusted alternative splicing constructs (Fig. 4) than *L. vannamei*, *Paralithodes camtschaticus* and *Panulirus ornatus* in its eyestalk, gill, hepatopancreas and muscle tissue despite it having the highest nominal expression (in terms of mapped reads) in nearly all the aforementioned tissues. The positions of Outrigger called splicing constructs could be mapped to 1870 unique transcripts in *B. latro*, 1586 in *L. vannamei* 1220 in *Panulirus ornatus*, and 1067 in *Paralithodes camtschaticus*. *Birgus latro* therefore has a lower absolute number of alternative splicing constructs but more unique transcripts under splicing than the decapod crustaceans it was compared to. The coconut crab seemingly makes up for a reduction in alternative splicing constructs with notably higher proliferation of individual genes compared to its counterparts (Table S6, Additional file 6). The ratio between the two main classes of alternative splicing constructs we report on – skipped exons (SE) and mutually exclusive exons (MXE) – seems to be characteristic of the respective species under study. MXEs are reported in the literature as a “rare subtype” [12], but we show that it is only in *B. latro* where SEs are clearly the dominant construct, with muscle and hepatopancreas in *B. latro* having SE:MXE ratios in excess of 30. The dominance of SEs is also more pronounced in *B. latro* than in *Paralithodes camtschaticus* and *Panulirus ornatus* in its eyestalk and gill tissue, but the dominance ratio in *B. latro* drops by an order of magnitude in these tissues (Fig. 4). MXEs not only seem to be more prevalent in general in the genomes that we studied, but they are also the dominant construct in *L. vannamei* gill and muscle tissue. Interestingly, a comparison of putative regulators of alternative splicing with detected homology to known sequences and more than 25% serine/arginine (SR) content reveals that the high SR-content proteins in *B. latro* is relatively less known than the *Panulirus ornatus* high SR-content proteins as is indicated by the ratio of known gene symbols to unknown genes symbols (Fig. 5).

Most genes with more than 100 copies in a given species, are most proliferated in *B. latro*. The most notable of these are *kif22* with 2402 copies followed by *tigd7* with 1827 copies. The coconut crab also shows proliferation of genes involved in the visual, respiratory, olfactory and cytoskeletal systems. We furthermore observed significant proliferation of the HOX gene, *caudal*, that is known to play a role in posterior development in *Drosophila* [13], but this feature could not be placed in the context of carcinization since *caudal* expansion is present in both the short-tailed anomurans and the long-tailed achelatan. Table S7 (Additional file 7) contains gene ontology (GO) annotations with descriptions under biological process, cellular component and molecular function for each of the significantly proliferated genes that had data available in Flybase or Entrez (under *Drosophila melanogaster*, *Homo sapiens*, *Danio rerio* or *Caenorhabditis elegans*).

Although the spiny lobster, *Panulirus ornatus*, has noteworthy gene proliferation of its own (e.g., *lanb1*, a protein that functions in the basement membrane), it is the undetected genes in *Panulirus ornatus* that are of particular interest, the most prominent of these being the *ybx1* and *ybx2* genes, which are abundantly expressed within the Anomura. With the exception of *ybx1,* that is also expressed in the true crab, *Portunus trituberculatus*, the *ybx* genes are exclusively present in the Anomura. The *ybx* genes are orthologously clustered with genes under the functional categories of translation and transcription. *Ybx1*, amongst other assigned GO annotations, plays a role in tRNA transport. The expansion of this tRNA transport gene in the Anomura and *Portunus trituberculatus* stand in contrast to the non-detection of a threonyl-tRNA synthetase, *aats-thr*, in *B. latro*. The pattern of non-detection of coding features in *Panulirus ornatus* is extended to its non-coding complement (e.g., *U3* snoRNA) and to the non-splicing of a gene, *wupA*, that is under heavy splicing in the Anomura (Fig. S1, Additional file 8). Lowering the detection threshold of non-coding features did not lead to a copy of the important non-coding *u3* snoRNA being detected in *Panulirus ornatus*. However, a copy of the coding *u3-55k* was detected in *Panulirus ornatus* with only *Armadillidium vulgare* and *Parhyale hawaiensis* also having copies of *u3-55k* (Table S8, Additional file 11). The u3-55k protein is known to interact with *u3* snoRNA [14] and it should be noted that non-detection of *u3* in the spiny lobster does not necessarily imply its absence. With the detected presence of *u3-55 k*, at least a variant of *u3 snoRNA* is therefore expected to be present in the *Panulirus ornatus* genome. In contrast to the spiny lobster, the *u3* non-coding gene is massively proliferated in both the blue and red king crabs (family Lithodidae). Two coding genes, *ddx11* and *zbed8*, likewise stand out for being massively proliferated in the Lithodidae. *Ddx11*, amongst diverse annotations, is associated with rRNA transcription, which points to a function that is complementary to that of the non-coding *u3*.

## Discussion

Of the three genomes that were assembled in this study, the genome of the coconut crab, *Birgus latro*, was the most interesting in terms of informativeness. The coconut crab has a high number of proliferated genes, seemingly in lieu of alternative splicing (see concept ideogram in Fig. S2, Additional file 9). This characteristic, that could plausibly be associated with terrestrialism, is not observed in the three aquatic decapods which the coconut crab was compared to. We start the following discussion with an elaboration on gene proliferation and alternative splicing with reference to plant regulation of alternative splicing in the context of terrestrialization. We then substantiate our hypothesis that the coconut crab uses alternative splicing as an adaptation mechanism, and finally, we point out limitations in our study and suggest a focus for future research to determine whether alternative splicing is indeed a terrestrial adaptation mechanism in the coconut crab.

### Muted alternative splicing in *Birgus latro* compared to fully aquatic decapods

**Fig. 6:**
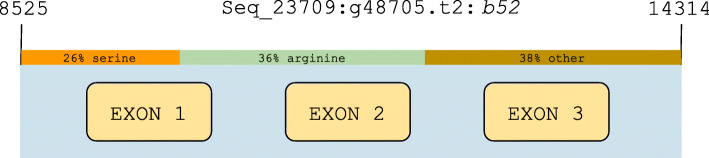
A gene structure ideogram of the high SR-content encoding *b52* gene. The *b52* gene contains three exons and an exceptionally high percentage of serine/arginine (SR) amino acids. This ideogram depicts the position of the gene in the *Paralithodes camtschaticus* genome. Besides from this gene’s product being exceptional high in SR-content, it is also of interest since it is present in all assembled genomes. This ideogram was drawn with Google Drawings

The alternative splicing profiles of the aquatic *Paralithodes camtschaticus*, *Panulirus ornatus* and *L. vannamei* show that there is higher reliance on alternative splicing in them than in the terrestrial *B. latro*. The muted alternative splicing in *B. latro* is pervasive across all tissue types assayed, and as previously mentioned, this is in spite of *B. latro* displaying a higher nominal expression in all tissues. The higher nominal expression does not appear to have an adverse effect that might distort this interpretation since the gills of a *L. vannamei* specimen from a salt-perturbation experiment (inferred from sequence read archive data [15]) shows higher nominal, absolute and reads-per-million adjusted alternative splicing than *B. latro* (Fig. 4). Gill and hepatopancreas tissue show similar clustering of high alternative splicing in the aquatic species in contrast to low alternative splicing in the terrestrial *B. latro*. This pattern is also observed in eyestalk tissue but seems to follow light availability, with the dark-dwelling red king crab, *Paralithodes camtschaticus*, undergoing the highest alternative splicing followed by the other two species that live in shallower waters where there is better light availability. The high level of splicing in low light availability habitats intuitively points to alternative splicing as an adaptation mechanism in response to light depravation. No data with regards to light regulated alternative splicing has been reported for *B. latro*, but the *dscam* gene, which is significantly proliferated in *B. latro* and that has been annotated with function within the visual system, has been reported as proliferated and alternative spliced in the semi-terrestrial Chinese mitten crab in an immunological context [16]. This expansion of a gene annotated with function in the visual system is an important shared characteristic between the coconut crab and the Chinese mitten crab since they both have adapted to the terrestrial environment where a higher reliance on vision is typically required. Interestingly, alternative splicing events are known to be induced by light signals in plants [17], and furthermore, changes in alternative splicing profiles are rapidly induced (within the hour) by changes in light conditions [18]. These studies suggest that the obligate terrestrial *B. latro* might have different alternative splicing profiles during its larval and juvenile stages when it navigates the sea and the salty beachfronts (see concept ideogram in Fig. S2, Additional file 9). The reason for the high proportion of SR-protein genes without a gene symbol in *B. latro* could indicate functional redundancy of these putative regulators of alternative splicing. Nevertheless, *B. latro* does exhibit alternative splicing in all its studied tissues, with one of the genes under splicing in all tissues being the highly proliferated *kif22*. Although we found a SR-content protein expressed from the *b52* gene (Fig. 6) notable for its exceptionally high SR-content and because it appears in the genomes of all three assembled genomes, it is not possible to draw a conclusion as to what extent alternative splicing in *B. latro* is regulated by SR-content proteins based on our data. To the best of our knowledge, comparative information on SR-protein regulation of alternative splicing is not available in animals, but studies on land adaptation in plants have associated proteins with high SR-content with alternative splicing in a tissue specific and stress responsive manner [19, 20] and it is hypothesized SR-proteins play a pivotal role in terrestrial adaptation in plants.

### Proliferated genes in *Birgus latro* overlap in function with tissues that exhibit muted alternative splicing

Clustering of orthologous coding sequences by functional categories reveals massive proliferation in *B. latro* of the previously mentioned cytoskeleton related gene, *kif22*, and a second gene, *tigd7*, tagged with cell cycle and chromatin structure in mammals. Numerous other genes are also most proliferated in *B. latro* when compared to the other species under study, but not to the extent that these two genes are. These less proliferated but still significantly proliferated genes in the *B. latro* genome include genes that function in the visual (e.g., *moe*, *ry*, *dscam*), respiratory (e.g., *moe*, *rbcn-3a*, *gammacop*), and muscular systems (e.g., *mhc2*). Genes tagged with microtubule related function (e.g., *moe*, *kif22*, *kifap3*), other cytoskeleton related genes less proliferated than *kif22* (e.g., *gammacop*), and genes tagged with mitochondrial function (e.g., *atp7*, *cyp2u1*, *drp1*, *sdha*) are also prominently proliferated in *B. latro*. Given that *B. latro* has atypical mitochondrial DNA [6] with notable mutations across its tDNA complement, the proliferation of genes tagged with microtubule motor function (e.g., *kif22*, *khc*) and organelle transport along the microtubule (e.g., *gammacop*) are of particular interest since nuclear protein-coding genes supplement mitochondrial function. Mitochondria are the powerhouses of the cellular environment and it therefore makes intuitive sense that the expansion of genes involved in microtubule functioning leads to the expression of proteins that affect cellular energy production in *B. latro* since microtubules are directly and indirectly associated with the cellular distribution of mitochondria [21]. This theme of expansion of nuclear genes with mitochondrial and microtubule related function is similar to the theme of gene expansion in lieu of alternative splicing and suggests that *B. latro* also underwent evolution in its mitochondrial DNA during its adaptation to the terrestrial environment (see concept ideogram in Fig. S3, Additional file 10). The energy cost of locomotion on land (with some caveats with regard to speed of locomotion) is known to be higher than that of locomotion in air and water [22]. *Birgus latro’s* relatively large size and awkward locomotion would therefore place a significant energy burden on it compared to an aquatic organism of the same size and speed of locomotion. This suggests that evolutionary adaptation to the land would have had to be accompanied by substantial genomic change in *B. latro*, especially in its genes that are involved in energy production.

As can be seen from the preceding examples, some genes such as *kif22*, *gammacop* and *moe*, have multiple functional categories and are recurring in examples across the functional categories of interest. These three genes are furthermore respectively annotated as functioning in organelle transport along the microtubule [23], in cuticle development [24], and in microtubule organization [25]. The aforementioned cuticle-associated coatomer gene, *gammacop*, may play a role in hardening of the adult coconut crab abdomen due to a need for greater cuticle sclerotization in a terrestrial environment [26]. As far as canonical developmental genes are concerned, only a single HOX gene, *caudal*, was found to be significantly proliferated in our study. However, our comparative gene expansion analysis revealed four significantly proliferated genes annotated as functioning within olfactory biological processes, namely: *nf1*, *nmdar1*, *shi* and *plexb*. *Plexb* is the only one of these genes that is most proliferated in *B. latro*. Previous studies have shown that the Coenobitidae have developed enlarged olfactory lobes [27], presumably to enhance olfactory reception in a terrestrial environment. Olfactory anatomy in amphibians is known to change with metamorphosis, which is in turn associated with migration from an aquatic to terrestrial environment [28].

### Hypothesis on the role of alternative splicing in *Birgus latro’s* terrestrial adaptation

Alternative splicing is a major source of protein diversity and it is under precise temporal regulation [29]. Cells can switch between expression of alternative gene constructs by, for example, post-translational modification of splicing factors [30] that are often high in serine and arginine (SR) residues. The role of these factors in alternative splicing regulation is well documented in plants [19, 20] and the hypophosphorylation of SR-proteins has been observed to inactivate splicer-dependent but not constitutive splicing [31]. Alternative splicing therefore has differing degrees of permanency. Plants had to overcome the barriers of desiccation, temperature fluctuation, and increased ultraviolet radiation on their way to adapting to life on land [32]. In contrast, while desiccation certainly remains an important barrier, land adaptation barriers in the Arthropoda are considered to be more related to reproduction, osmoregulation, locomotion and sensory reception [2]. We argue that the knowledge gained from SR-content studies in plants can nevertheless be applied to the study of alternative splicing regulation in crustaceans since the property of high SR-content is a physicochemical property. As shown in our results, the Anomura has a disproportionate number of high SR-content proteins with homology to known sequences but without identifiable gene symbols. This suggests that the high SR-content complement of the Anomura is less well known, and furthermore that studies on this specific protein complement in decapods are needed to determine whether there exists a regulatory role for SR-proteins in alternative splicing in crustaceans as has been shown to exist in plants. A shortcoming in our assessment of alternative splicing is that we only had a single RNA-seq replicate of each tissue type for each assembled genome since our RNA-seq data was originally intended to assist with functional annotation of the genomes. However, given that the coconut crab displays lowered alternative splicing in all four of its tissue types, the concern is rather about biological repeats than it is about technical repeats. Despite a shortage of literature on the regulation of alternative splicing by SR-proteins in animals and our single RNA-seq technical replicate per tissue type, we have revealed that the coconut crab follows a pattern of low alternative splicing across all the studied tissues and that there exists within the coconut crab highly proliferated gene sets that overlap in function with the function of the tissue types. We have furthermore observed differences in alternative splicing profiles based on publicly available *L. vannamei* RNA-seq data, which raises the question as to whether the coconut crab has a different alternative splicing profile during its development in the aquatic environment, and later on, during its time as a juvenile in the intertidal zone. The best way to answer this question would be to obtain the transcriptomes of coconut crab zoeae, glaucothoes and juveniles under conditions that mimic the respective in situ habitats. In other words, experimentation should be extended to include both tissue (spatial) and life stage (temporal) data. This approach is beyond the scope of the present study. Such work would however provide a definitive answer as to what extent alternative splicing in the coconut crab is dynamically regulated over the course of its life.

## Conclusion

The brevity of gene expansion examples for *Paralithodes camtschaticus* and *Panulirus ornatus* compared to the ample examples of features of interest in the *B. latro* genome emphasizes the extent to which it stood out during comparison to its contextual peers. The coconut crab distinguishes itself in terms of (1) genomic repetitive content, (2) taxonomic classification of homologs, (3) functionality assigned by orthologous clusters and gene ontology, (4) alternative splicing, (5) mitochondrial-targeting sequence signals, and (6) gene expansion. We suggest that the coconut crab’s remarkably different genomic content has provenance in the most notable of differences that separates *B. latro* from the other decapods under study – its habitat. Our results have shown that *B. latro* has proliferated genes with functions that overlap with those of the tissue types in which it has less alternative splicing constructs than other decapods, and secondly, it has proliferated cytoskeletal and mitochondrial related genes that may act to supplement atypical mitochondrial tDNA. The first ever draft nuclear genome of the coconut crab reported here has provided a first genome wide glimpse into terrestrialism in the ecological niche of the Coenobitidae, and furthermore provides a resource for the generation of hypotheses for future genomics studies on topics such as hermit crab shell-utilization.

## Methods

### Sample collection and whole genome sequencing

The adult *Panulirus ornatus* and *Paralithodes camtschaticus* specimens (one each) were purchased from the Tai Po Fish Market in Hong Kong, with the former caught from local waters and the latter from fishery catch in Alaska and imported to Hong Kong. The single *Birgus latro* specimen was purchased from the First Makishi Public Market in Okinawa, Japan. All animals were transported alive to The Chinese University of Hong Kong and acclimatized for 2 weeks before dissection for tissue isolation, which was carried out after putting the animals on ice until they became immobilized. High-molecular-weight DNA was extracted from muscle tissue using Qiagen Genomic-tip 20/G (Qiagen, Hilden, Germany). For each species, paired-end libraries with insert sizes of 350 bp were constructed using a Truseq PCR Free Kit following manufacturer’s instructions. Sequencing was conducted using an Illumina Hiseq 10X platform with a read length of 150 bp. Chromium 10X libraries were constructed for each species according to manufacturer’s protocols and sequenced on an Illumina Hiseq 10X platform (for *B. latro*) and a Novaseq-6000 platform (for *P. ornatus* and *P. camtschaticus*) with a 150 bp read length. Refer to the NCBI projects listed in the data availability section for a full listing of read libraries.

### Genome assembly

The 10X linked-read files, consisting of two pairs of read files for the king crab, three pairs for the coconut crab and eight pairs for the lobster were decontaminated using Kraken version 2.0.8b [33] with its default database. The decontaminated linked-read files were then de novo assembled using Supernova version 2.1.1 [34] without performing any further pre-assembly filtering steps on the linked-read files as per assembler guidelines. Supernova automatically estimates genome size, and we report these estimates for each studied species. Next, raw assembly output files were converted from binary format to pseudo-haploid fasta format using the *mkoutput* algorithm bundled with Supernova. Gaps in the assembly were subsequently filled with the Illumina 2 × 150 paired-end short-reads using Baseclear’s Gapfiller version 1.10 [35] after adapter trimming and base error correction was carried out on the short-reads using Fastp version 0.20.0 [36]. Duplicate contigs were removed from the gap filled assemblies using the BBmap [37] dedupe algorithm. Summary assembly statistics were generated with the Assemblathon [38] stats algorithm. Finally, genome completeness was assessed with BUSCO version 3.0.1 [39] using arthropod database version odb9. *Drosophila melanogaster* was selected as the model species for training Augustus [40] during the execution of the BUSCO pipeline. All genomes were preprocessed for further analysis using wrapper scripts from the funannotate pipeline [41]. Preprocessing included sorting assembly fasta files by contig size, renaming the contigs to simpler names, and removing contigs shorter than 200 bp. Repetitive elements within the genomes were identified and soft-masked with Repeatmasker version 4.0.7 [42] using de novo models created with RepeatModeller version 1.0.11 [43–46].

### Species for comparative genomics

High-quality published genomes of four decapods were chosen for comparative genomics: the Pacific white shrimp (*Litopenaeus vannamei* [47];), the marbled crayfish (*Procambarus virginalis* [48];), the swimming crab (*Portunus trituberculatus* [49];) and the blue king crab (*Paralithodes platypus*; GenBank: GCA_013283005.1). An updated version of the blue king crab genome was later published by Tang et al. (2021) [50]. The isopod *Armadillidium vulgare* [51] and the amphipod *Parhyale hawaiensis* [52] were furthermore included as outgroups. Genomes for the species used for comparison were either downloaded from online repositories, or in the case of *L. vannamei*, obtained directly from the authors. Predicted protein sequences could not be obtained for *Paralithodes platypus* and were therefore predicted using Genemark-ES and an Augustus helper script from the Braker2 pipeline (see next section).

### Structural genome annotation

Structural annotation of coding elements was carried out using the Braker version 2.1.4 pipeline [53–59]. The Braker pipeline was used for ab initio prediction as well as for RNA-seq assisted prediction using bam files generated by STAR-aligner version 2.7.3a [60]. Transcriptomic data for four tissue types of the three target species were obtained from the crustacean annotated transcriptome (CAT) database [61] and used for RNA-seq assisted prediction. These tissue types were: hepatopancreas (ovary instead of hepatopancreas for the spiny lobster), eyestalk, gill and muscle. A Spearman rank correlation of the aligned RNA-seq reads of all tissue types was obtained using Deeptools version 3.3.1 [62]. The genome annotation generator (GAG) version 2.0.1 [63] was used to annotate start and stop codons missed by Braker2, to remove exons shorter than 15 bp as well as introns and coding sequences shorter than 10 bp, and to summarize the structural annotation results. Structural non-coding feature annotation was carried out using Rfam database version 13.0 [64] with infernal version 1.1.2 [65]. Non-coding features were stringently filtered to include only those with E-values smaller than 1.0 × 10^− 5^. For more specific information on tRNAs, Rfam-predicted tRNA sequences were extracted from the genomes with Bedtools version 2.29.2 [66] and filtered to exclude duplicates using Genometools version 1.5.10 [67]. The extracted and filtered sequences were then passed as input to tRNAscan-SE [68]. Various output files produced by software programs employed in our study were parsed and tabulated using RStudio version 1.1.456 [69] with the R packages *data.table* version 1.12.8 [70] and *plyr* version 1.8.6 [71]. HOX gene homologs within predicted protein data of *Paralithodes camtschaticus*, *Panulirus ornatus* and *B. latro* were identified using a comprehensive *D. melanogaster* homeobox gene symbol list [72]. Variants within the genomes were called using GATK version 4.1.7.0 [73] after preprocessing steps that included the use of BWA version 0.7.17-r1188 [74] Samtools version 1.7 [57], and Parallel version 20,161,222–1 [75].

### Transcript abundance determination and alternative splicing profiling

We assessed alternative splicing as a secondary goal since we had RNA-seq data available to carry out such an assessment. Raw transcriptomic reads were sourced from the CAT database [61] for *Panulirus ornatus*, *Paralithodes camtschaticus* and *B. latro*, and from GenBank’s sequence read archive (SRA) for *L. vannamei* (SRR9208110, SRR12113701, SRR1737225, SRR1951371, SRR6466295 and SRR2060963). The raw paired RNA-seq reads for muscle, hepatopancreas (not for *Panulirus ornatus*), gill, eyestalk, and ovary (last tissue only for *Panulirus ornatus* and *L. vannamei*) were mapped back to the genomes using STAR aligner version 2.7.3a to quantify transcript abundance per gene. Alternative splicing profiling was subsequently carried out using Outrigger version 1.1.1 [76]. Candidate alternative splicing constructs were identified if they were flagged by Outrigger as having sufficient forward and reverse reads mapped to their constructs (the so-called “case 8” assigned by Outrigger), and if the splicing event had a percent spliced-in (psi/Ψ) value greater than 0.05. Relative expression of alternative splicing constructs was calculated for constructs that could be mapped to known gene symbols as well as unknown gene symbols (structural annotation identifiers were used in the latter case). The datasets were standardized by tissue type per species so that the most highly expressed construct in each tissue could be emphasized with color coding.

### Functional genome annotation

Peptide sequences (translated from predicted coding sequences) were used as input to Eggnog version 5 [77] to search for homologous peptides and to group peptides by functional categories assigned to orthologous clusters. In addition, we divided putative genes with detected homology into two groups based on whether they had gene symbols associated with them or not. We used the relative sizes of the two groups to each other as a gauge for how well the homologs have been studied by the scientific community. Peptide sequence output from Braker2 was also scanned for mitochondrial targeting sequences using TargetP version 2.0 [78], after which peptides flagged as mitochondrial targeting peptides (mTPs) where scanned with Eggnog version 5. All organisms’ peptide sequences were used to identify single-copy genes, shared orthologs, and to infer gene/species trees using Orthofinder version 2.3.11 [79] with the FastTree version 2.1.10 inference algorithm [80] selected as the tree inference algorithm. Single-copy orthologue groups were then individually aligned using MAFFT version 7.402 [81] with the “auto” setting, and ambiguous parts of the alignments were trimmed by trimal version 1.4.rev15 [82] using the “gappyout” option. The best partitioning scheme and substitution model were selected by IQtree version 1.6.12 [83], and we used the same program to conduct maximum likelihood tree construction with 1000 ultrafast bootstrap replicates. As gene proliferation may indicate that a gene confers an advantage on the organism in its environment, we carried out gene expansion analysis with Café version 5 [84–86] using predicted homologs with known gene symbols for coding and non-coding gene sets, as well as for amino acid anticodons, and for the set of HOX genes. For each of the four preceding sets, Café was executed with filtered subsets that differed in copy number by less than 100 in order to allow successful execution of a data convergence step in the Café algorithm. Genes that were excluded from Café analysis by the filtering process were manually retrieved using R scripts, which also mitigated the exclusion of genes by Café itself based on the requirement that at least one copy of a given gene should be present in the basal outgroup. Molecular function, biological process, cellular component, and accompanying GO terms associated with significantly proliferated genes, were retrieved using *MyGene.info* [87]. Software used to visualize results in this study include Circlize version 0.4.10 [10], UpSetR version 1.4.0 [11] and ggtree version 2.2.3 [9].

## Supplementary Information

**Additional file 1: Table S1.** Structural genome annotation summary.

**Additional file 2: Table S2.** tRNA amino acid anti-codon bias.

**Additional file 3: Table S3.** Best taxonomic classification of coding sequences with known homologs.

**Additional file 4: Table S4.** KOG/COG functional categories (with key at bottom of spreadsheet).

**Additional file 5: Table S5.** Single copy orthologs present in all nine organisms.

**Additional file 6: Table S6.** Coding and non-coding gene expansion analysis.

**Additional file 7: Table S7.** GO annotations with biological concepts for all genes identified in expansion analysis (refer to GO ontology for evidence codes)

**Additional file 8: Figure S1.** Heatmaps of the most highly alternatively spliced genes per tissue type. Alternative splicing profiles of genes with known gene symbols (top) as well as genes without known gene symbols (bottom) for (A) *Birgus latro*, (B) *Paralithodes camtschaticus* (C) *Panulirus ornatus* (D) *Litopenaeus vannamei*. The *wupA* gene is noteworthy due to it being spliced in all species except *Panulirus ornatus*. These heatmaps were drawn with RStudio version 1.1.456 [69] (with R base packages) and Microsoft PowerPoint.

**Additional file 9: Figure S2.** The concept of gene expansion in lieu of alternative splicing. We observed a proliferation of genes with annotated functions that overlap with those of the tissues where the coconut crab has lower alternative splicing than its aquatic counterparts. Based on our observation that there are differing alternative splicing profiles for the gills of *L. vannamei*, we hypothesize that the coconut crab might have a higher reliance on alternative splicing during its time in the marine environment. This ideogram was drawn with Google Drawings.

**Additional file 10 Figure S3.** The concept of nuclear expressed gene expansion supplementing atypical mitochondrial DNA. A previous study has shown that the coconut crab’s mitochondrial tDNAs are notably mutated compared to other anomurans. In the present study, we observed within the nuclear genome of the coconut crab highly proliferated genes that are annotated with mitochondrial and microtubule function, and a massively proliferated kinesin, *kif22*, that has the most mitochondrial targeting signals within its homologous gene sequences compared to other decapods. This points to the nuclear expressed protein complement having a more prominent supplementary role with respect to the mitochondrion in the coconut crab. This ideogram was drawn with Google Drawings.

**Additional file 11: Table S8.** Eggnog assigned gene symbols.

## Data Availability

Curated versions of the genomes assembled, annotated and analyzed during the current study are available from the Zenodo repository (DOI: 10.5281/zenodo.4589425). NGS reads that were used to assemble these genomes are available under the following NCBI BioProjects: PRJNA704570 (*Birgus latro*), PRJNA704576 (*Panulirus ornatus*) and PRJNA704614 (*Paralithodes camtschaticus*). Transcriptomic datasets not generated but relied on in this study are available at the NCBI’s SRA repository for *L. vannamei* (SRR9208110, SRR12113701, SRR1737225, SRR1951371, SRR6466295 and SRR2060963), and from the authors of the crustacean annotated transcriptome (CAT) database [61] for the prior three organisms. Access rights and login credentials to the aforementioned transcriptomic data was granted by the authors of the CAT database, and the aforementioned transcriptomic reads were used with their permission. The remainder of genomes used for comparative purposes are publicly available for *Litopenaeus vannamei* (GenBank: GCA_003789085.1) [47], *Procambarus virginalis* (http://marmorkrebs.dkfz.de/downloads/genome/pvirGEN-0.4/) [48], *Portunus trituberculatus* (http://gigadb.org/dataset/100678) [49], *Paralithodes platypus* (GenBank: GCA_013283005.1) [50], *Armadillidium vulgare* (GenBank: GCA_004104545.1) [51], and *Parhyale hawaiensis* (GenBank: GCA_001587735.2) [52]. Except for the access controlled transcriptomic database administrated by the authors of the CAT database, all the other aforementioned data are publicly available on open access terms.

## Abbreviations

GO: Gene ontology
LINE: Long interspersed nuclear element
LTR: Long terminal repeat
mTP: Mitochondrial targeting protein
MXE: Mutually exclusive exon
PSI: Percent spliced in
SE: Skipped exon
SINE: Short interspersed nuclear element
SR: Serine-arginine
