# Supplementary material for: Comparative genomics of the coconut crab and other decapod crustaceans: exploring the molecular basis of terrestrial adaptation
Source: BMC Genomics. 2021 Apr 30;22:313. doi: 10.1186/s12864-021-07636-9 (PMC8086120; doi:10.1186/s12864-021-07636-9)
Supplement: Supplementary file 8 — Additional file 8: Figure S1. Heatmaps of the most highly alternatively spliced genes per tissue type. Alternative splicing profiles of genes with known gene symbols (top) as well as genes without known gene symbols (bottom) for (A) Birgus latro, (B) Paralithodes camtschaticus (C) Panulirus ornatus (D) Litopenaeus vannamei. The wupA gene is noteworthy due to it being spliced in all species except Panulirus ornatus. These heatmaps were drawn with RStudio version 1.1.456 [69] (with R base packages) and Microsoft PowerPoint. [file 12864_2021_7636_MOESM8_ESM.pdf]

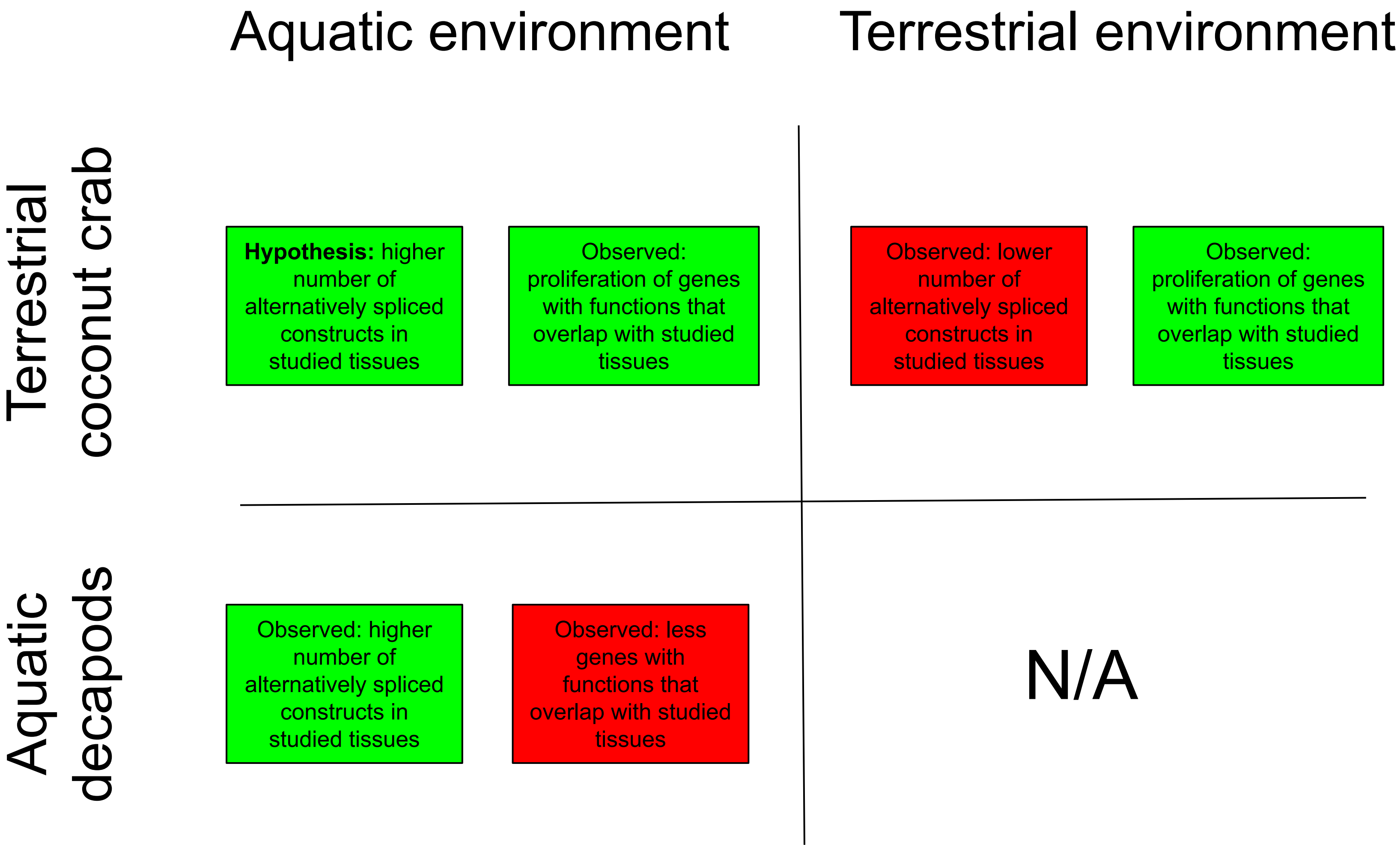
Aquatic environment
Terrestrial environment
Terrestrial coconut crab
Aquatic decapods
Hypothesis: higher number of alternatively spliced constructs in studied tissues
Observed: proliferation of genes with functions that overlap with studied tissues
Observed: lower number of alternatively spliced constructs in studied tissues
Observed: proliferation of genes with functions that overlap with studied tissues
Observed: higher number of alternatively spliced constructs in studied tissues
Observed: less genes with functions that overlap with studied tissues
N/A
