# Supplementary figures and images for: Comparative genomics of the coconut crab and other decapod crustaceans: exploring the molecular basis of terrestrial adaptation
Source: BMC Genomics. 2021 Apr 30;22:313. doi: 10.1186/s12864-021-07636-9 (PMC8086120; doi:10.1186/s12864-021-07636-9)

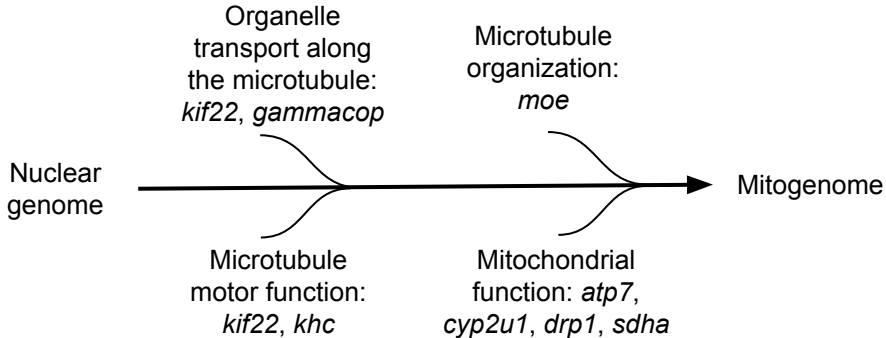

Supplement: Supplementary file 9 — Additional file 9: Figure S2. The concept of gene expansion in lieu of alternative splicing. We observed a proliferation of genes with annotated functions that overlap with those of the tissues where the coconut crab has lower alternative splicing than its aquatic counterparts. Based on our observation that there are differing alternative splicing profiles for the gills of L. vannamei, we hypothesize that the coconut crab might have a higher reliance on alternative splicing during its time in the marine environment. This ideogram was drawn with Google Drawings. [file 12864_2021_7636_MOESM9_ESM.pdf]

TOP

A

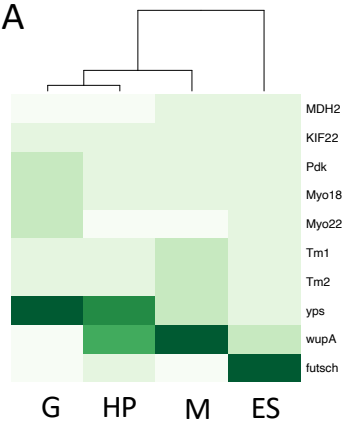

B

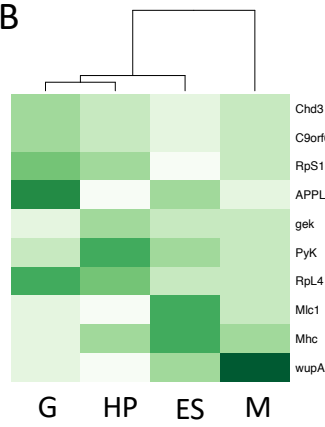

C

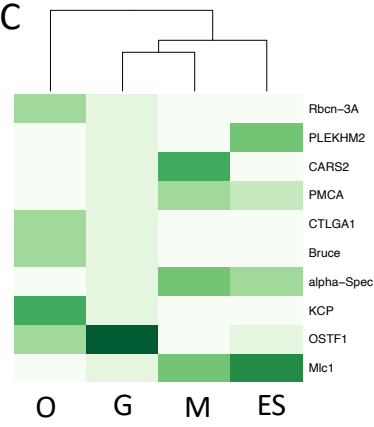

D

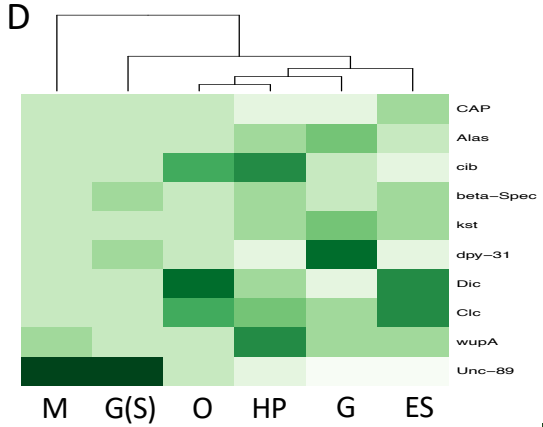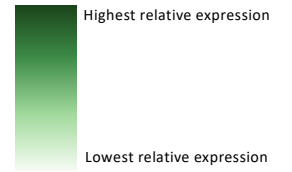

BOTTOM

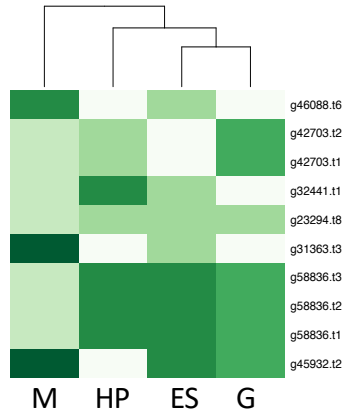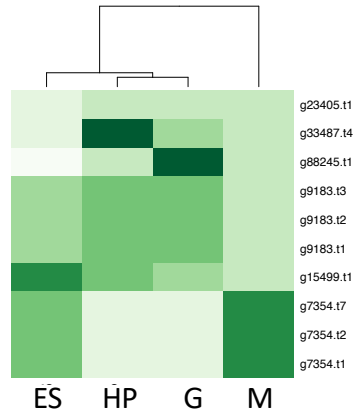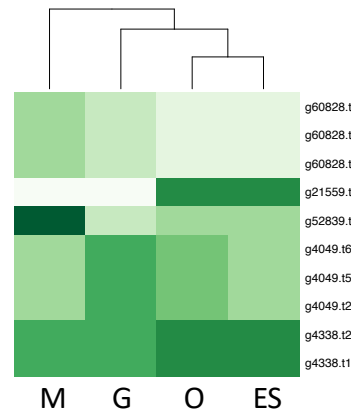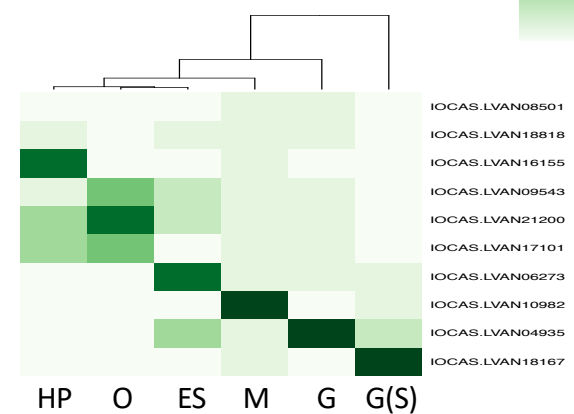

ES: Eyestalk   G: Gill   G(S): Gill (salt perturbed)   HP: Hepatopancreas   M: Muscle   O: Ovary

Supplement: Supplementary file 10 — Additional file 10 Figure S3. The concept of nuclear expressed gene expansion supplementing atypical mitochondrial DNA. A previous study has shown that the coconut crab’s mitochondrial tDNAs are notably mutated compared to other anomurans. In the present study, we observed within the nuclear genome of the coconut crab highly proliferated genes that are annotated with mitochondrial and microtubule function, and a massively proliferated kinesin, kif22, that has the most mitochondrial targeting signals within its homologous gene sequences compared to other decapods. This points to the nuclear expressed protein complement having a more prominent supplementary role with respect to the mitochondrion in the coconut crab. This ideogram was drawn with Google Drawings. [file 12864_2021_7636_MOESM10_ESM.pdf]
